# At the heart of genetic disease: an interview with Elizabeth McNally

**DOI:** 10.1242/dmm.041566

**Published:** 2019-09-18

**Authors:** Elizabeth McNally

**Affiliations:** Elizabeth McNally is Director of the Center for Genetic Medicine at Northwestern University's Feinberg School of Medicine. She has directed an independent laboratory for more than 20 years and mentored more than 80 trainees.

**Keywords:** Genetic disease, Interview, Neuromuscular disease

## Abstract

Elizabeth McNally is a human geneticist and a cardiologist whose research has been instrumental in understanding the mechanisms of inherited heart and skeletal muscle diseases. She is the Director of the Center for Genetic Medicine at Northwestern University's Feinberg School of Medicine. In this interview, Elizabeth talks about her first experiences in science, the importance of understanding complex genetic interactions and the effort we all need to make to facilitate diversity in science.


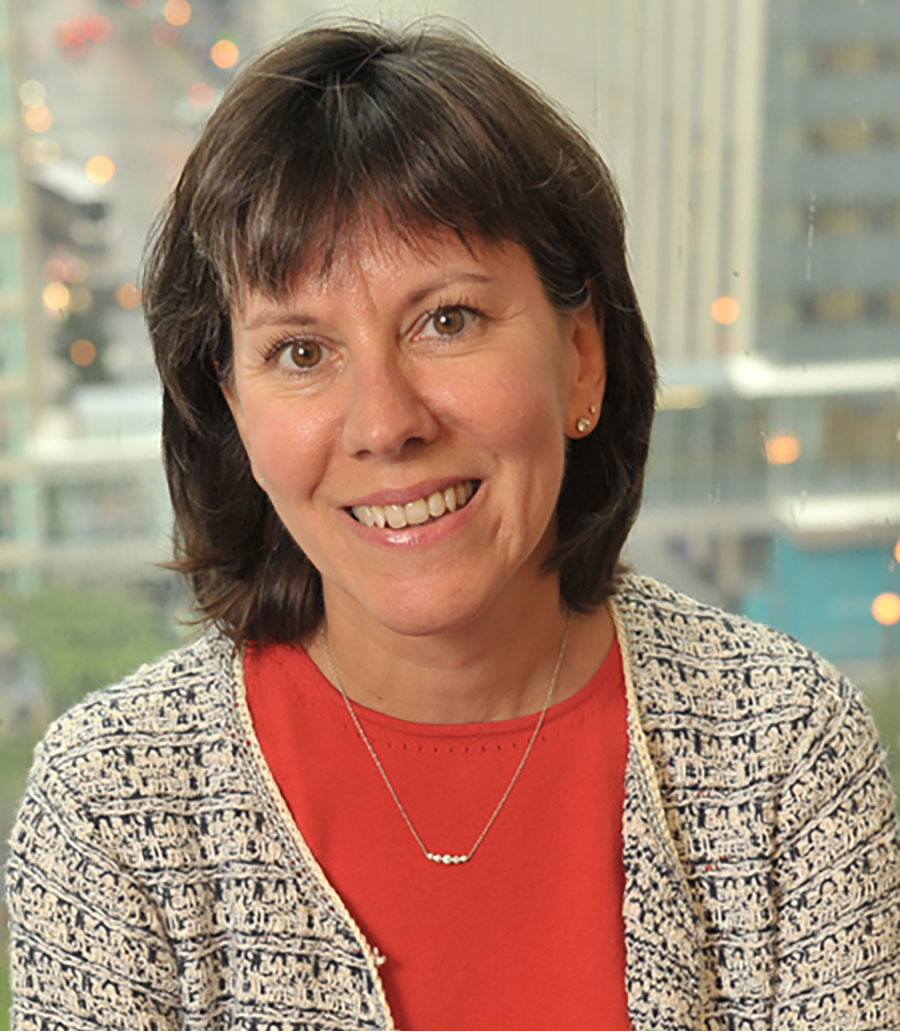


**Introduction**

Elizabeth McNally received her MD and PhD degrees from Albert Einstein College of Medicine under the mentorship of Leslie Leinwand [currently Chief Scientific Officer of the University of Colorado Boulder's BioFrontiers Institute] and completed postdoctoral training at Harvard with Louis Kunkel. Elizabeth studies genetic disorders that affect cardiac and skeletal muscle. As a physician-scientist at Northwestern's Feinberg School of Medicine, she and her team define disease mutations in patients, use model systems to elucidate their function and translate this knowledge into effective therapies. Her group's identification of the modifying effects of a TGFβ binding protein on the fragility of the muscle membrane and on muscle fibrosis is leading to the development of a therapeutic antibody for patients with Duchenne muscular dystrophy and other muscle-wasting diseases.

**Did you always want to be a scientist?**

I came at it a little differently, I think. From a pretty young age I was interested in medicine and wanted to be a physician. I always say that a major reason I eventually became a scientist was because there is a wonderful science museum here in Chicago, the Museum of Science and Industry, and I loved visiting it as a kid. But I never knew there were actual scientists [laughs], because I was never exposed to any growing up. It wasn't until college that I came to meet a few scientists and as soon as I saw what they did I knew that was what I was going to do. So I was always interested in medicine, but I didn't realize that there were people doing both science and medicine.

**Could you tell us a bit more about how you came to focus on cardiology and on the genetics of neuromuscular disease?**

When I was an undergraduate, I was very fortunate to spend a summer working in a research lab and got hands-on exposure. At the time, molecular biology was in its infancy and the only way we could look at human DNA was through Southern blotting. The lab I was working in was one of the first and very few with expertise in this method. My project was to identify mutations in muscle genes in patients with muscle disease. This was before PCR was invented and before the human genome was sequenced, so you can imagine how ridiculous the idea was that we would find something relevant by Southern blotting. The concept of how much genomic sequence difference there would be from person to person was very fuzzy. We knew about mutations, of course, but we really had no understanding of the depth of the person-to-person differences outside of mutations. We were going at it blind, but it was exciting for me. The general approach, using genetic disease information to better understand how the world works, had potential. If we could figure out how the genes work in disease, we could infer a great deal as to how the proteins work normally.

**Could you share the key lessons that you learned during that summer?**

It was really interesting and a formative experience, because as I said, I had almost no prior exposure to how science works. I was very lucky to work with a fantastic technician who taught me, a young student, how to do things. Nowadays, I always say to my trainees that the best thing about science is that you can start doing it right away, but the only way the process really works is if you have someone who teaches you how to do it. And once you learn, you find yourself teaching the next person. So my lesson to first-year students is that they have to ask others for help and then eventually pay it forward – a year later, they'll be helping other people. I love this hands-on process as much as I love the overarching concept of what we're doing.

**An important focus of your lab is the study of genetic modifiers and epistasis. What are the main challenges in unveiling these complex relationships?**

There are so many challenges because there are so many genetic modifiers. So I think one of the main ones is the vastness of this network. We know very well that genes are not the only modifiers of outcome or phenotype. There are environmental factors – for muscle and heart disease, exercise, diet, tobacco smoke and other factors are huge modifiers of outcome. But those are much harder to track and model. So in my lab, where we work with model systems like cells, flies and mice, and we can control their environment, for example by controlling the source and composition of the food, to homogenize the background which enables us to tease out the genetic modifiers of a phenotype or outcome. The really hard part is that there are so many modifiers, so even when we do detailed quantitative trait locus mapping and we identify a locus, it remains entirely possible that there is more than one single genetic modifier within that locus. So we focus as much as possible on identifying plausible candidates, defining specific genetic variation and then elucidating how that variant affects the primary disease gene or process. We find it's necessary to support these genetic observations with functional assessments of what the genetic modifiers may be doing. At least by understanding the mechanism, we can learn more about the disease process more broadly and about the variants involved.

**Can you tell us more about how we could apply our understanding of epistasis to extrapolate general regulatory principles and to develop novel treatments of muscle diseases?**

Our whole approach is to solve modifiers to uncover biological pathways that might be useful for therapy development. The first two or three hits that we identified pointed to an overarching pathway, and the first modifier we mapped was an incredibly strong modifier affecting both the fragility of the muscle membrane as well as the amount of muscle fibrosis or scarring. We were able to measure these two quantitative traits and map them to a TGFβ binding protein called latent TGFβ binding protein-4 (LTBP4). What was cool about this story is that the genetic change in *LTBP4* pointed exactly to the molecular domain that regulates binding to TGFβ. So the sequence variant immediately told us that that part of the molecule was functionally important. So now we are working with a company to develop antibodies against that domain, as these antibodies will hold LTBP4 in a conformational state that inhibits latent TGFβ release, and ultimately this can be used as a therapeutic in patients. So that's probably our biggest home run so far, because both the genetics and the functional testing worked out so beautifully, the variant had a powerful effect. It was especially nice that genetic variation in the same gene in humans also had the same modifier effect. So, everything stacked up to what we were hoping for in this process, and that is precisely the reason why we do this: we hope that the genetics will teach us about the mechanism and that we can go on to exploit this mechanism to develop an effective treatment.

The second modifier we mapped brought a much more detailed understanding of what the muscle membrane repair complex looks like, and it's giving us very good ideas for a therapeutic agent that we're currently developing in collaboration with another company. These modifiers are prompting us to think about therapeutics because they help us to really understand the mechanism of how a certain disease works.

“… that is precisely the reason why we do this: we hope that the genetics will teach us about the mechanism and that we can go on to exploit this mechanism to develop an effective treatment.”

**How did you navigate the process of bringing your research from the lab to a company?**

It was actually a really fun and interesting five years. We published the first LTBP4 paper about ten years ago, and we realized pretty quickly that it was an obvious drug target. We were lucky that our discoveries coincided with the pharmaceutical industry's increased interest in rare disease, so the timing was on our side. We approached the University of Chicago, where I was based at the time, but the University, not knowing as much about it as we did, was not interested in pursuing the idea. Luckily, they released the intellectual property to me, and I was able to pursue it along with a former postdoc and grad student. Rather fortuitously, another of my former postdocs is now an intellectual property lawyer, and his knowledge was very helpful with the patent application. The patent allowed us to seek additional funding because intellectual property is the currency in these ventures. This first endeavour was a great learning opportunity for me, and I now understand that academia and industry are actually complementary and very powerful together.

I have had a few trainees in the lab who are interested in pursuing a career in industry, and I try to share what I learned with them to help them prepare for this transition. I also included my trainees as co-inventors on the patents, because I think this is very important. I really believe that everyone who contributes should get credit.

**Your work involves several model systems, and we know that some genetic interactions may be different in different species. What do you think researchers need to consider when studying epistasis in animal models?**

I think one of the most important things we need to learn is whether the modifiers we identify are directly acting on the disease process. We work on forms of muscular dystrophy in which the muscle membrane is very fragile, constantly undergoing damage and repair. So we need to consider the regenerative aspect of the tissue in these diseases. This is an area where it is tough to completely understand how well insights from animals translate into humans, because we don't yet know at which stage of the human disease the regenerative potential of the muscle diminishes or whether it does really wear out over time. We have great data from zebrafish, mice and fruit flies, but we know that the baseline regenerative potential in healthy mice, fish or flies differs and is probably quite different from that of humans.

**Could you tell us a bit about what the main drawbacks and advantages of such multi-platform research are?**

Many [laughs]. We try to keep abreast with the technical developments in animal models. Our core experience is with mice, as we spent a lot of time working with mouse models. We do less work in fly models and, at any given time, we typically have just one or two people in the lab who work with flies. So they need to have a supportive community of *Drosophila* people outside of the lab to interact with and adopt the latest tools and techniques from. Fortunately the fly community is lively and there are great meetings that my lab members can attend. And we have excellent collaborators on campus here at Northwestern. In general, keeping up with so many new and emerging technologies is the hardest part. Like many labs interested in modelling different genetic diseases, we are increasingly working with human cells, which adds its own set of complexities. For humans in particular, who are all so different from each other, deciding what is considered a ‘normal background’ or baseline cell is difficult and will vary from study to study. Is the patient-derived cell the best for study, or is it the CRISPR-engineered version in which a mutation has been inserted into a ‘normal’ background? We don't yet have a good answer to that.

And the scientific community seems to keep raising the bar all the time. Now, it's not unusual to see a human cell model alongside a mouse model in the same paper. This can be hard for the trainees, because we live in a time when we can ask big questions but it takes so long to get answers.

**Can you tell us more about the translational potential of cell-based models of muscular diseases, such as iPSC-derived myotubes?**

These models have their limitations, some of which I alluded to before – the genetic variability of patient-derived cells and the lack of a ‘consensus’ well-characterized control. We know a patient's genetic background will contribute to their disease, but we do not yet understand it fully. The second big drawback is that induced pluripotent stem cell (iPSC)-derived cells don't fully mature into the cell types we are trying to study. They enter a differentiation programme, but do not reach its terminus. There are physiological aspects of, for example, heart and skeletal muscle cells, like calcium handling, that are very different in a fully mature cell and are therefore really hard to replicate in a stem cell-derived model. But that doesn't mean that these models are not useful. Many of us in the genetic disease field are recognizing their potential for personalized medicine. We can model an individual patient's disease genetics and test therapeutics such as exon-skipping antisense oligonucleotides, gene editing or antibodies and thus devise a potentially effective treatment relatively quickly. But this treatment may apply to only one person in the world with that mutation. Interestingly, from a regulatory standpoint, we don't yet have any means of applying this personalized treatment to a patient because the regulators don't know how to deal with this ‘*n*=1’ approach to drug development. But we recognize that, in five to ten years, this will change dramatically if the scientific community, regulators and patient groups come up with the right solutions.

**At the 2018 American Society of Human Genetics meeting, Timothy Yu from Boston Children's Hospital presented a recent case in which they identified a unique Batten disease mutation in a single patient and went on to develop the custom exon skipping oligo (which he named after the patient), tested it and obtained approval to administer it to the patient within a year. Many of the patient's symptoms stabilized. This is an example of the ‘*n*=1’ drugs you mentioned**

Nowadays we occasionally see families that have the drive and the means to contact the relevant researchers and maybe they are able to raise the necessary funds, so there are occasional cases like the one you mentioned. It is encouraging, as these families are providing a blueprint pathway from genetic diagnosis to drug. But this path shouldn't be limited to families that know how to ‘work the system’. As geneticists, we see the problem and now we even have some tools to fix it. Hopefully these fixes will soon be accessible to more patients. In the past five years since we've witnessed this explosion of personalized medicine, we've also seen increased interest from drug companies for what the solutions would look like.

**Switching gears now. I noticed that the majority of your team, both close collaborators and trainees, are women. Was this by design?**

It mostly just happened. I think many established women scientists would agree that we get many more women applicants for jobs. I am not entirely sure why that is, maybe having a supervisor of the same sex provides a level of comfort. It's important that we recognize this, because I think it could also explain why the reverse is true, with many male-led labs ending up being full of men. And although this may make us more comfortable, it's also a source of the bias that we need to work against to ensure diversity. We had occasions when we had to aggressively recruit men to make sure the lab is balanced. So the lesson is that you can't just let things happen, you have to work towards a balanced representation. And once we have achieved this balance, we need to continue working to ensure that the responsibilities and opportunities are shared equally.

When I started my postdoc with Lou Kunkel, I was among his first female trainees and I remember one of the most annoying things was that it defaulted to the women to answer the phone. When it rang, the guys would just wait for one of us to answer, until we decided we'd just let it ring. It drove us crazy [laughs], but it was a small example one of the many things that we need to consciously decide to correct.

**As a woman in a leadership role, are there any measures to support women and other underrepresented groups that you are particularly mindful of?**

It think it requires constant effort. At this stage of my career, I can provide opportunities for younger women at different career stages and I go out of my way to make sure we invite women and minority colleagues for seminars and interviews. I'm encouraged by the fact that current trainees, PhD students and postdocs are an incredibly diverse group, and we need to make sure we retain them, mentor them and ensure success. Because that's what the world currently looks like and it's important that the leadership looks like the group that it's leading. That's the message I try to live by and my lab is currently incredibly diverse. But it requires constant work, we can't count on it happening naturally. We constantly need to make efforts to counteract bias.

“Current trainees are an incredibly diverse group, and we need to… retain them, mentor and ensure success. Because that's what the world currently looks like and it's important that the leadership looks like the group that it's leading.”

Although awareness is improving, I think most academic medical centres are lagging a bit behind in bringing diversity to the top positions, which are mostly held by white men. The reasons for this are complex and we need to understand at least some of it to effect meaningful and lasting change. Often leaders are recruited from outside an institution, and outside recruitment may be harder for women. Some brilliant women may find it hard or are unwilling to move. I'm a fan of giving opportunities to inside candidates that we've groomed and supported within our own institution. In certain areas, we're achieving good diversity balance amongst trainees and junior faculty, but there's a dramatic drop-off beyond the assistant professorship level that we need to address. I think ensuring tailored support for these diverse junior faculty and hiring good people, even internally for senior roles, is a perfectly good way of both ensuring excellence and bringing diversity to academic leadership. It's a bit like gardening – it requires constant tending.

**Are there any pearls of wisdom you'd like to share with your junior colleagues?**

I think everyone should put more emphasis on communicating their science. When talking about your work, it's important to know your audience and to know what you are pitching. So many people are doing amazing work, but it doesn't mean anything if people don't know about it. Whether sharing it through the publication process or in an elevator or to the lay public or to lawmakers, it's important to know how to talk about your work. We, as a community, probably don't spend enough time learning how to communicate our science, so we need to put more emphasis on it.

**And finally: what do you enjoy doing outside of the lab?**

I'm a huge sports fan. I was an athlete when I was younger, and I enjoy the competitiveness of sports. In the summer I watch a lot of baseball, and in the winter, it's basketball. NBA finals is one of my favourite times of the year because I get to enjoy a lot of good basketball and not think about work as much. I always support local teams, I'm probably one of the few female season ticket holders for the Chicago Bulls and I support the Sky, which are our local WNBA team.

